# Editorial: Multiomics and multiparametric analyses to characterize myeloid cell subsets

**DOI:** 10.3389/fimmu.2023.1292400

**Published:** 2023-09-20

**Authors:** Thien-Phong Vu Manh, Marc Dalod, Diana Dudziak

**Affiliations:** ^1^ Aix-Marseille University, CNRS, INSERM, CIML, Centre d’Immunologie de Marseille-Luminy, Turing Center for Living Systems, Marseille, France; ^2^ Laboratory of Dendritic Cell Biology, Department of Dermatology, Friedrich-Alexander University of Erlangen-Nürnberg (FAU), University Hospital Erlangen, Erlangen, Germany; ^3^ Deutsches Zentrum Immuntherapie (DZI), Friedrich-Alexander University of Erlangen-Nürnberg (FAU), University Hospital Erlangen, Erlangen, Germany; ^4^ Comprehensive Cancer Center Erlangen-European Metropolitan Area of Nuremberg (CCCER-EMN), Friedrich-Alexander University of Erlangen-Nürnberg (FAU), University Hospital Erlangen, Erlangen, Germany; ^5^ Friedrich-Alexander University of Erlangen-Nürnberg (FAU), University Hospital Erlangen, Erlangen, Germany; ^6^ Institute of Immunology, Jena University Hospital, Friedrich-Schiller-University, Jena, Germany

**Keywords:** myeloid cells, multiomics, multiparametric analysis, cell type identification, cell state characterization

Myeloid cells are heterogeneous innate leukocytes of bone marrow origin, endowed with crucial roles for proper immune system functioning. They encompass distinct cell types: granulocytes (neutrophils, eosinophils, basophils, mast cells), monocytes, macrophages and dendritic cells (DCs). Each of them has distinct roles: for instance, neutrophils rapidly respond to infections by engulfing and destroying various pathogens; macrophages promote tissue remodeling, repair and maintenance, including clearing cellular debris, pathogens and foreign particles; DCs excel in antigen processing and presentation, orchestrating adaptive immunity. Moreover, myeloid cells are functionally plastic, which ensures host adaptability to evolving challenges: depending on the pathophysiological context, each myeloid cell type can undergo different activation states associated with distinct activities. This has been well studied for macrophages whose phenotypes and functions are shaped by their tissue niches (1) and by inflammatory cues (2).

Considering their importance in all aspects of immunity, myeloid cells are interesting targets to improve treatments against cancer and other difficult-to-treat diseases including autoimmune or inflammatory pathologies, allergies and certain infections. However, to target the right cell type and activation state depending on the pathophysiological context, efficient and safe harnessing of myeloid cells in the clinic requires better characterizing their identity, functions and molecular regulation. This task is being revolutionized by multi-omics and multiparametric analyses that have recently opened a new era of deep, and possibly unbiased, characterization of biological samples, including analyses at the single-cell level (2). This Research Topic aims at illustrating this revolution, while also providing state-of-the-art examples of computational analyses of single myeloid cell omics datasets.

Rigamonti et al. investigated the heterogeneity of human circulating monocytes, consensually classified based on CD14 and CD16 expression. CD14^+^, CD14^+^CD16^+^ and CD16^+^ monocytes are called classical, intermediate and non-classical monocytes, respectively. Single-cell transcriptomics analyses unveiled nine monocyte populations, each poised for specialized functions. Classical monocytes segregated into states linked to inflammatory, neutrophil-like, interferon-related or platelet-related pathways. Non-classical monocytes encompassed two states, with one harboring high expression of complement components. This insight was applied to diseases, using a machine learning-based classification, revealing shifts in monocyte populations/states in advanced cancers, post-immunotherapy and COVID-19, suggesting their differential engagement in various pathologies.

In another chapter, Kao et al. focused on gastric myeloid-derived suppressor cells (MDSCs). MDSCs encompass different cell types and states, which must be considered to determine their physiological roles. Indeed, inaccurate resolution of this heterogeneity may confound associations between cell types/states and functions. The heterogeneity of gastric MDSCs was analyzed by single-cell RNA sequencing (scRNAseq), with a Cxcr2+ subset identified and shown to regulate gastric pathology linked with lipid peroxidation dysregulation.

Tiemeijer et al. investigated macrophage functional heterogeneity for cytokine secretion upon TLR4 stimulation, using an in-house single-cell droplet-based approach. One macrophage subset produced both IL-10 and TNF, without displaying other obvious differences with non-IL-10 producers. Macrophage density modulated the dynamics of their TNF and IL-10 production, highlighting the significance of the communication between individual macrophages in the modulation of their population-wide responses to tissue inflammation (quorum sensing).

Lang et al. harnessed scRNAseq to study how inflammasome signaling works in the myeloid populations associated with Head and Neck Squamous Cell Carcinoma (HNSCC). Different clusters of tumor-infiltrating myeloid cells expressed distinct inflammasome-related genes. NLRP3 and IL-1β transcripts were enriched in specific clusters of monocytes or macrophages. Deleting NLRP3 decreased tumor progression *in vivo*, recapitulating the effect of caspase-1/IL-1β inactivation. Efferocytosis of apoptotic tumor cells was shown to activate myeloid-intrinsic NLRP3 dependent inflammasome signaling in the tumor microenvironment, hence promoting tumor growth via IL-1β secretion.

Single-cell transcriptomics was used by Podojil et al. to decipher how targeting myeloid cells using advanced nanotechnology can improve treatments against cancer. Poly(lactic-co-glycolic acid) nanoparticles, called ONP-302, were previously reported to ameliorate inflammatory diseases via myeloid cell targeting. Here, this approach was applied to a murine melanoma model. ONP-302 infusion restored anti-PD-1 receptiveness in refractory tumors, which was associated with triggering of the cGAS/STING pathway in myeloid cells, leading to enhanced NK cell activation via IL-15, and increased PD-1/PD-L1 expression in the tumor microenvironment.

Gouin et al. harnessed scRNAseq to investigate the dynamical responses of lung cell types to *ex vivo* lung perfusion (EVLP), a system that enhances donor lung suitability for transplantation. Datasets from human lungs declined for transplantation and maintained in EVLP for 4 or 10 hours were analyzed using advanced computational methods for unbiased cell type identification. Inflammation-related pathways were activated in specific cells, including monocyte-derived and alveolar macrophages at 4-hours. Cytoskeleton signaling pathways were reduced in various cell types, particularly at 10 hours. CD4^+^ and CD8^+^ T cells, NK cells, mast cells and conventional DCs displayed blunted activation at both time points, suggesting that, despite inducing inflammation, EVLP might improve lung transplantation outcome by dampening various immune cell type activation.

To examine the impact of ApoA4 deficiency on liver immune cells in a mouse model of nonalcoholic fatty liver disease (NAFLD), Liu et al. used single-cell transcriptomics. Mice deficient for ApoA4 harbored increases in specific cell types, including inflammatory macrophages and activated granulocytes, with altered gene expression profiles. Key ApoA4 targets including IL-1β were confirmed to drive the reprogramming of liver immune cells. Thus, ApoA4-responsive immune cells and pathways represent interesting therapeutic targets to treat NAFLD.

A computational pipeline was developed by Patel et al. to facilitate deep and unbiased analysis of complex, high-content, data from flow cytometry, mass cytometry and multiplexed immunofluorescence. This pipeline performs dimensionality reduction, clustering, evaluation and optimization of clustering resolution, and also provides downstream visualization tools. Developing such open-source and user-friendly software for the analysis of high-dimensional multi-parameter data is of paramount importance to empower researchers with diverse backgrounds to efficiently uncover meaningful insights without having to immerse into complex coding endeavors.

In conclusion, this Research Topic illustrates how multiomics and multiparametric analyses are boosting our ability to dissect the complexity of myeloid cells. These types of approaches will be key to continue mapping myeloid cell types and their activation states in various pathological conditions, in order to determine their functions and molecular regulation, which is paving the way for developing novel strategies for precision medicine.

## Author contributions

T-PVM: Writing – original draft, Writing – review & editing. MD: Writing – review & editing. DD: Writing – review & editing.

## References

[1] Guilliams M Thierry GR Bonnardel J Bajenoff M. Establishment and maintenance of the macrophage niche. Immunity (2020) 52(3):434–51. doi: 10.1016/j.immuni.2020.02.015 32187515

[2] Bassler K Schulte-Schrepping J Warnat-Herresthal S Aschenbrenner AC Schultze JL. The myeloid cell compartment-cell by cell. Annu Rev Immunol (2019) 37:269–93. doi: 10.1146/annurev-immunol-042718-041728 30649988

